# Geographical Pattern of Malignant Neoplasm by Cluster Analysis Using Standardized Mortality Ratios (SMRs) in Ibaraki Prefecture, Japan

**DOI:** 10.2188/jea.12.143

**Published:** 2007-11-30

**Authors:** Setsuko Kinoshita, Hideto Takahashi, Masafumi Okada, Hiroaki Nishikawa, Satoshi Toyokawa, Katsumi Kano

**Affiliations:** 1 Graduate School of Medical Sciences, University of Tsukuba.; 2 Institute of Community Medicine, University of Tsukuba.; 3 Graduate School of Medicine, University of Tsukuba.; 4 College of Medical Technology & Nursing, University of Tsukuba.

**Keywords:** geographical clustering of cancer mortality

## Abstract

We investigated the geographical patterns of mortality from eight (males)/ten (females) sites of malignant neoplasm, using cluster analysis with Standardized Mortality Ratios (SMRs), and examined the relationship between the mortality structure and urbanization. To explore the geographical tendencies is important for the prevention of cancers; such as noticing risk factors associated with regional variance. The death rates, by site, gender and age from 1990 to 1994 in Japan, were obtained from Vital Statistics. The deaths and population in municipalities were obtained from “Population of Ibaraki Prefecture”. These were represented as averaged values in five-year periods. As an indicator of urbanization and mortality structure, the population density of municipalities and the overall rank scores of SMRs were used, respectively. Cluster analysis formed some distinctive structures. For males, Cluster 1 included four municipalities and three of these were located in the mountainous area in northwest Ibaraki, characterized by high SMRs from bone marrow. Cluster 5 consisted of the mid-south areas, characterized by high SMRs from stomach cancer. For females, the clusters seemed to be characterized by SMRs from esophagus cancer. An association between mortality structure and urbanization was found for females, 0.364(p<0.01), but not for males, 0.162(p=0.14).

## INTRODUCTION

Mortality rate caused by malignant neoplasm has been increasing in Japan, and malignant neoplasm has been the leading cause of death in Ibaraki Prefecture since 1985 where rapidly urbanized cities and large agricultural areas offer varied environments to residents^1^^)^.

Understanding the mortality pattern is necessary to analyze the cause of differences, in finding further unknown risks by examining numerous factors, such as the environment, geographical features, climate, eating habits and heredity.

The above studies are important to the determination of etiology and the development of preventive medicine^2^^,^^3^^)^. Errezola et al^4^^)^ suggested that the relationship between the geographical mortality of malignant neoplasm and the level of urbanization or industrialization should be researched. Noticing differences of risk factors between urbanized and rural areas advantages more sophisticated preventions that depend on actual conditions. Cluster analysis, called as unsupervised automatic classification in the field of the pattern recognition, is one of the statistical tools of the data analysis. Historically, this method has been utilized in the fields of education and psychology, and has also been adopted in epidemiology, especially in exploratory analysis. Tampieri et al^5^^)^ used cluster analysis as a descriptive method for studying an association between geographical pattern and mortality. The characteristic of the geographical pattern is discussed with other epidemiological findings. To investigate the characteristic of the geographical pattern on mortality from malignant neoplasm in Ibaraki Prefecture is an interesting problem. The purpose of this study is to analyze the geographical pattern in Ibaraki Prefecture using SMRs, as defined distance (dissimilarity), by cluster analysis and explore the association between the geographical pattern and the urbanization of municipalities.

## MATERIALS AND METHODS

### Data Source

Classification of malignant neoplasm followed by ICD-9, where the ten sites/types of malignant neoplasm (excluding breast and uterus cancer in males) in this study were the esophagus, 150; stomach, 151; rectum, 154; liver, 155; pancreas, 157; lung, 162; breast, 174; uterus, 179-182; leukemia, 204-208; and others. Site and gender specific deaths from malignant neoplasm and population with five-year age categories in each municipality in Ibaraki Prefecture for a five-year period between 1990 and 1994 were obtained by “Population of Ibaraki Prefecture”^6^^)^. The subject of the number of death is the one that has the legal domicile in Hokkaido, Honshu, Shikoku, Kyushu and Okinawa. The number of population is based on a census. The subject of a census is the one that dwells in Japan, excepting a diplomatic mission, a stuff of a consul and army members, and their family. The mortality rate of Japan, as a standard population, divided into five year age categories on each site of malignant neoplasm, was obtained by Vital Statistics^7^^)^.

### Statistical Methodology

SMR was calculated using the whole of Japan as a standard for each municipality. We used hierarchical cluster analysis with complete linkage method. Similarity between two municipalities is measured by Euclidian distance, d(x, y). For two municipalities x=[x_1_ ,x_2_, …, x_n_ ] and y=[y_1_ ,y_2_ , …, y_n_ ] are variations of “n” kinds of malignant neoplasms (males, n=8; females, n=10). Similarity is defined by d(x, y)= {∑_i_ (x_i_−y_i_)^2^ }^1/2^. The closest distance means that similarity is largest, in other words dissimilarity is smallest, and thus municipalities are combined and later clusters are combined until a final stage is reached where all municipalities are members of a single group^8^^,^^9^^)^. The software package S-PLUS 4.0 was employed for cluster analysis. To seek the relationship between SMRs and urbanization, we used quintile ranks. This ranking, which was suggested by Charlton, Hartley, Silver and Holland^10^^)^, was classified with scores, ranging from 1 (SMR is within the lowest 20% of all) to 5 (SMR is within the highest 20%). The representative value was given by the sum of these scores over sites of malignant neoplasm (overall rank score). As an indicator of urbanization, we used population density for all municipalities. To examine the affects of urbanization on SMR, Spearman’s rank correlation coefficient between overall rank score and population density was computed.

## RESULTS

Table 1 shows the variation in SMRs among municipalities of Ibaraki Prefecture, by sites of malignant neoplasm and gender. Miwa, Nanakai and Gozenyama had maximum SMRs values from several malignant neoplastic sites. Figures 1-1 and 1-2 show the dendrograms resulting from cluster analysis for 86 municipalities. The ratio of the least maximum distance of five clusters (males divided by females) was 0.73. The least maximum distance between clusters means the closeness of similarity. If this distance is small, then clusters are integrated earlier, so municipalities based on SMRs are characterized closer. The population density of an area, which was far from other clusters, showed a tendency of being low. The five clusters are shown as the mortality maps by gender in Figure 2-1 and 2-2.

**Figure 1-1.**
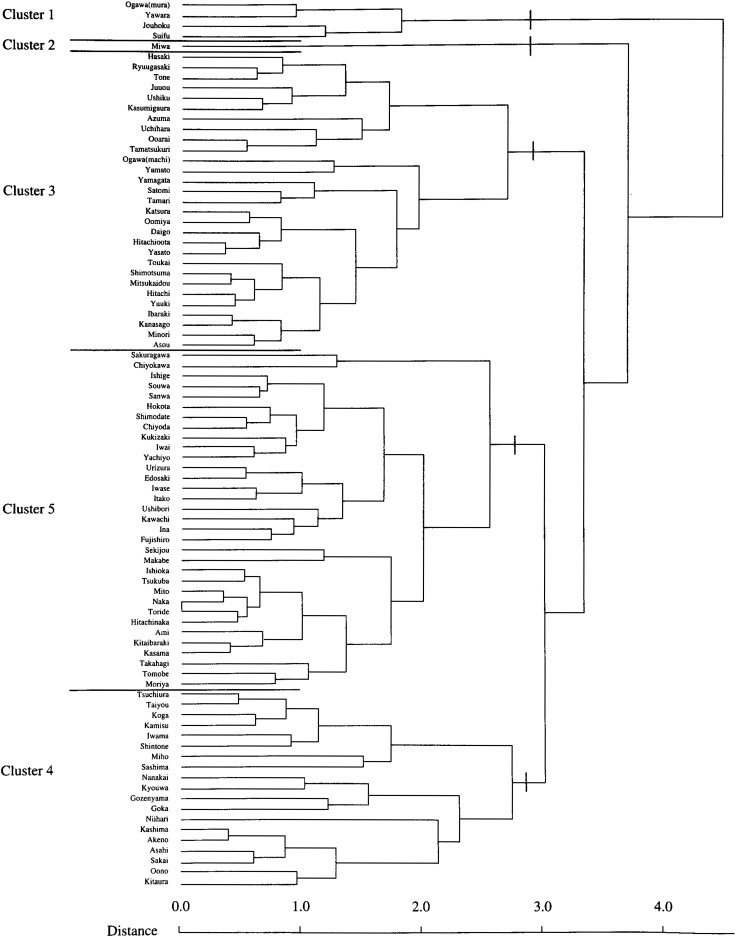
Dendrogram for 86 muicipalities on SMRs from malignant neoplasm for males (Ibaraki Prefecture).

**Figure 1-2.**
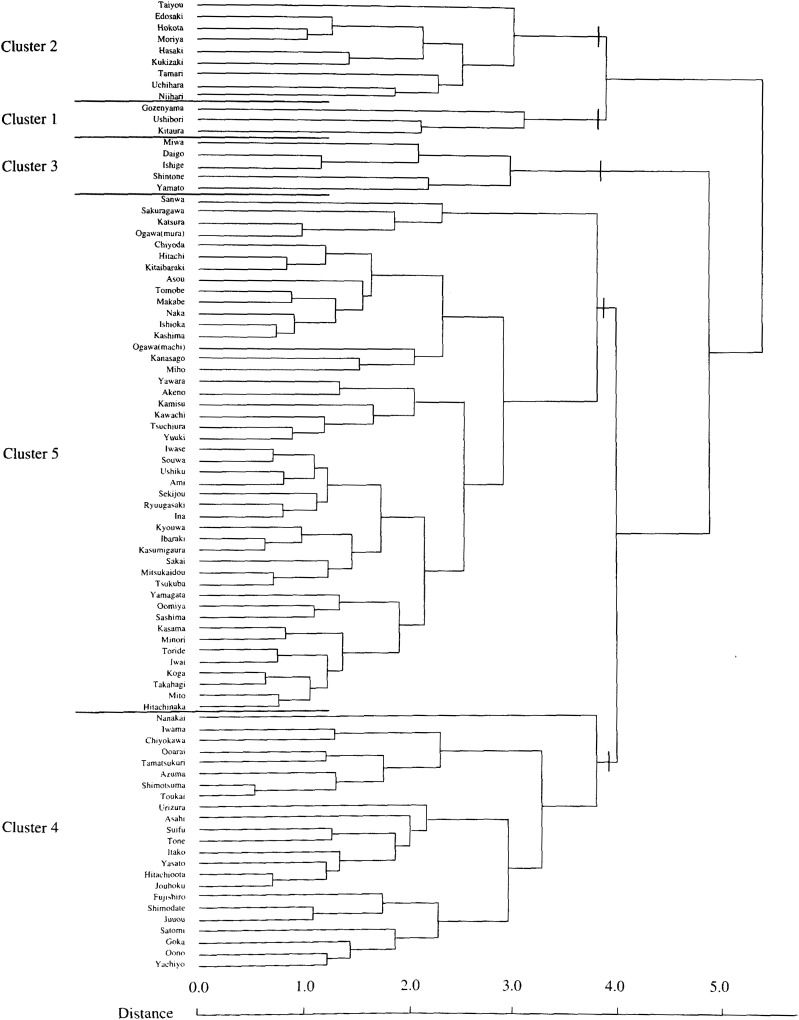
Dendrogram for 86 municipalities on SMRs from malignant neoplasm for females (Ibaraki Prefecture).

**Figure 2-1.**
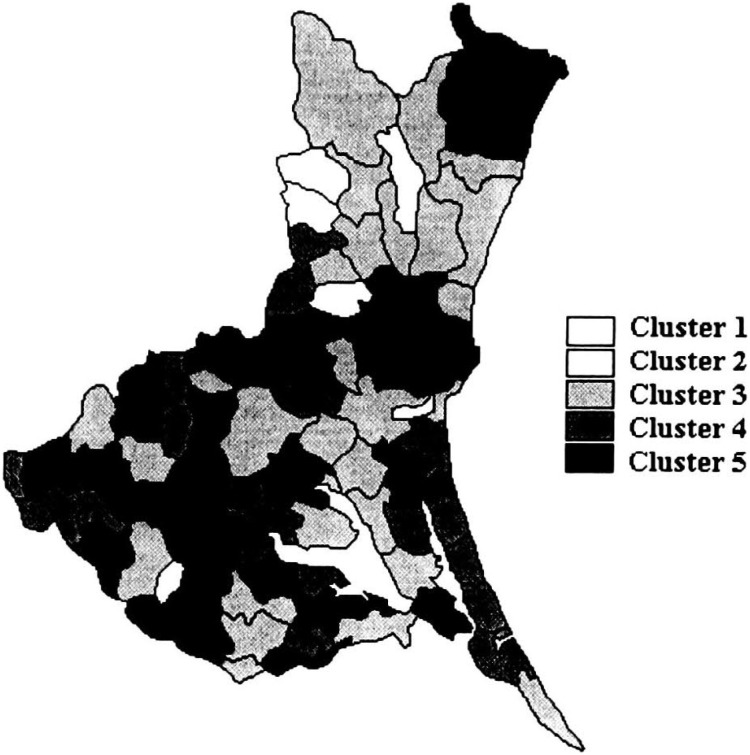
Mortality structure by five clusters for males (Ibaraki Prefecture).

**Figure 2-2.**
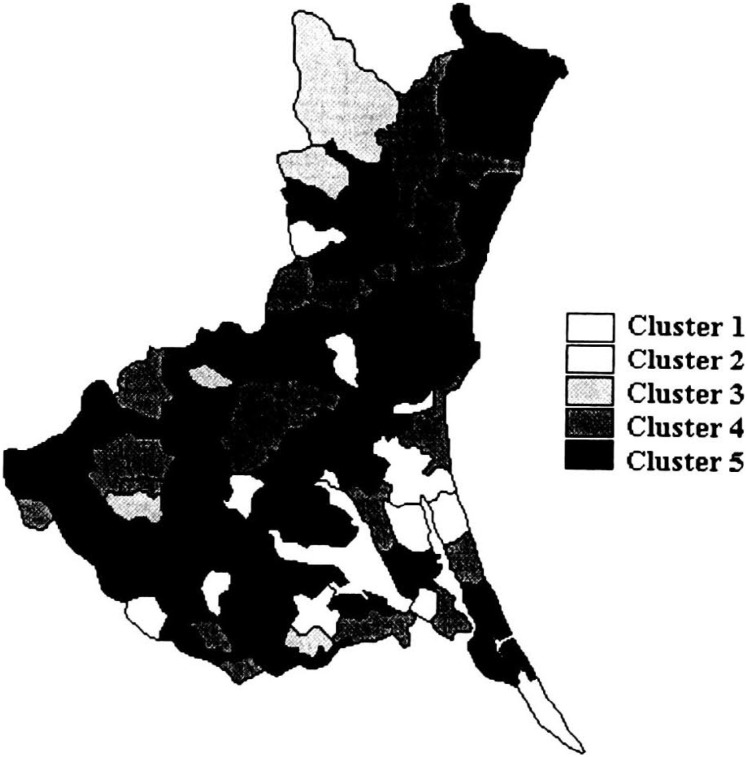
Mortality structure by five clusters for females (Ibaraki Prefecture).

**Table 1.** Fundamental statistics of SMRs by site (Ibaraki Prefecture, 1990-1994).

Site	Gender	Mean	SD*	Maximum**	Municipality	Minimum**	Municipality
Esophagus	Males	1.11	0.48	3.34	Miwa	0.33	Shintone
	Females	1.06	0.98	3.93	Yamato	0.00	28 regions
Stomach	Males	1.19	0.28	1.86	Makabe	0.46	Yamato
	Females	1.18	0.30	2.33	Miwa	0.53	Yamagata
Colon	Males	0.91	0.42	2.11	Niihari	0.00	Tamari
	Females	0.88	0.54	2.55	Miwa	0.00	11 regions
Liver	Males	0.76	0.36	2.33	Miura	0.19	Yamagata
	Females	0.77	0.36	1.85	Kamisu	0.00	2 regions
Pancreas	Males	0.96	0.44	2.50	Sakuragawa	0.00	Yamato
	Females	1.02	0.50	2.58	Chiyokawa	0.00	Nanakai
Lung	Males	0.91	0.24	1.77	Nanakai	0.40	Katsura
	Females	0.77	0.38	1.72	Tamatsukuri	0.00	3 regions
Breast	Females	0.86	0.48	2.51	Gozenyama	0.00	5 regions
Uterus	Females	1.05	0.60	3.40	Taiyou	0.00	4 regions
Bone marrow	Males	0.93	0.75	3.35	Yawara	0.00	11 regions
	Females	0.82	0.71	3.11	Gozenyama	0.00	20 regions
Others	Males	0.92	0.19	1.46	Ushibori	0.36	Satomi
	Females	0.97	0.24	2.21	Nanakai	0.50	Uchihara

SD*: Standard deviation; maximum/minimum SMR**; These values are followed by the corresponding location as listed.

Cluster 1 for males consisted of only four municipalities. The SMRs from leukemia in these municipalities were more than 3.0. Cluster 2 for males consisted of Miwa where SMRs from esophagus cancer were significantly high 3.3 (p<0.01). Cluster 1 could have been characterized by leukemia and Cluster 2 could have been characterized by esophagus cancer. Cluster 3 for males consisted of 29 municipalities and 12 of those had significantly low (p<0.01) SMRs from liver cancer. Cluster 4 for males, which consisted of 19 municipalities, was slightly ambiguous, but SMRs from colon cancer were likely to be high and SMRs from leukemia, low. Cluster 5 for males consisted of 33 municipalities and SMRs from stomach cancer were significantly high (p<0.05) for 13 municipalities.

Figure 2-1 (male mortality map) illustrates that the areas of Cluster 5 were mainly located in the mid-south of Ibaraki Prefecture, excepting Kitaibaraki and Takahagi. Figure 3-1 illustrates all areas with significantly high SMRs from stomach cancer for males. All municipalities except Kitaibaraki were located in the southwest of Ibaraki Prefecture.

**Figure 3-1.**
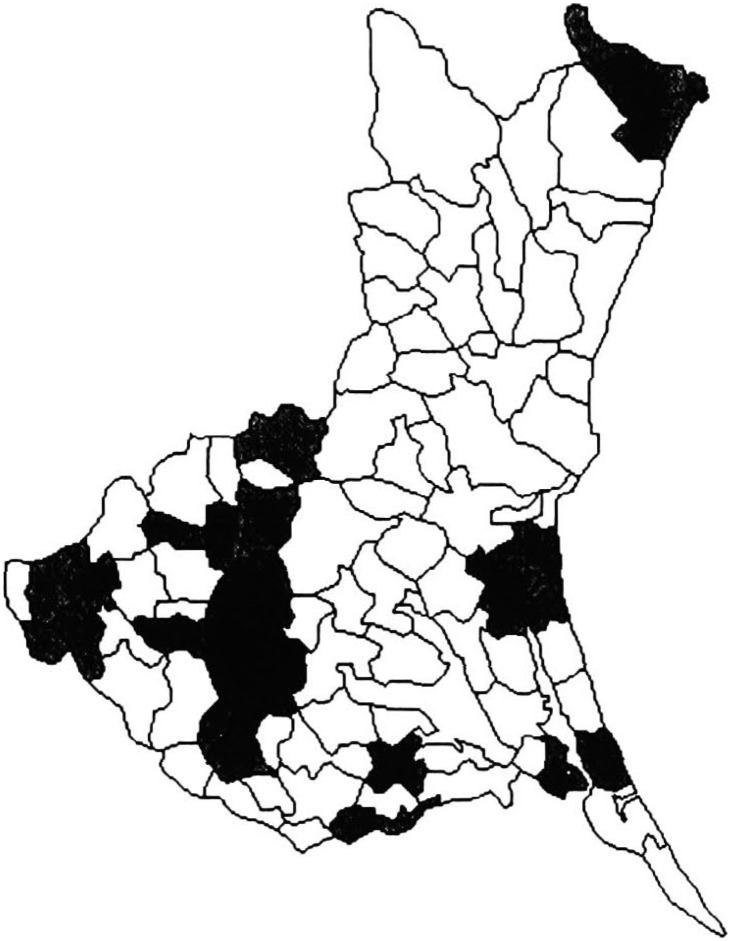
Areas of SMR, which are significantly high (SMR>1), from stomach cancer for males (Ibaraki Prefecture).

Cluster 1 for females contained three municipalities, Gozenyama, Ushibori and Kitaura, where SMRs from uterus cancer and leukemia tended to be high and esophagus cancer were low. Cluster 2 for females consisted of 9 municipalities where SMRs from pancreas and uterus cancers tended to be high and esophagus cancer were low. Clusters 3 consisted of 5 municipalities where SMRs from esophagus cancer were high and SMRs from breast and uterus cancers tended to be low. Cluster 4 consisted of 23 municipalities, where SMRs from esophagus cancer were low in 20 of 23 municipalities. Cluster 5 consisted of 46 municipalities where SMRs of esophagus cancer tended to be high. Figure. 2-2 illustrates the mortality map for females in which no obvious distinction was observed. The clustering could be characterized by whether the SMRs from esophagus cancer were 0 or not. Clusters 1, 2 and 4 comprised areas in which SMRs were mainly 0. Clusters 3 and 5 consisted of areas with generally high SMRs from esophagus cancer. In the cluster analysis for females, almost 50% of the municipalities in Ibaraki Prefecture belonged to Cluster 5. Figure 3-2 shows all areas of SMRs that exceeds 2.0 from esophagus cancer. The clustering of those areas was hardly observed.

**Figure 3-2.**
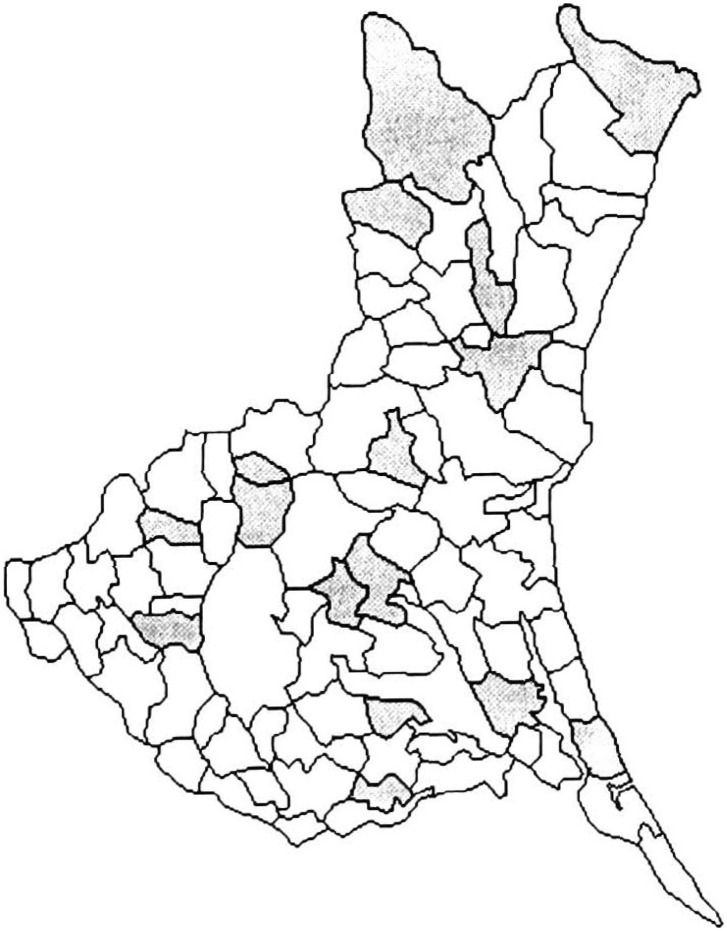
Areas of SMR, which are high (SMR>2), from esophagus cancer for females (Ibaraki Prefecture).

Spearman’s rank correlation coefficient between the overall rank score on SMRs and the population density was found for females, 0.364 (p<0.01), but not for males, 0.162 (p=0.14).

## DISCUSSION

Geographical pattern of malignant neoplasm in Ibaraki prefecture has been studied before, but never utilizing cluster analysis. Advantage of cluster analysis is that municipalities of similar mortality pattern are classified into a same group. In this study, geographical pattern shows that municipalities, which belong to a same group, are under similar circumstances of mortality from malignant neoplasms. For example, when malignant neoplasm is analyzed by one-dimensional SMR, we can get that lung cancer is high in the south area, or liver and pancreas cancer are high in the southeast area. On a contrary, by high-dimensional SMR, we can get which municipalities are under similar circumstances for mortality. The result was that mid-south area and northern 2 municipalities are under similar circumstances for males and municipalities of similar circumstances for females are dispersed throughout Ibaraki Prefecture. Our study utilizing cluster analysis revealed interesting characteristics, not by one-dimensional SMR but by high-dimensional SMRs.

To investigate etiology from malignant neoplasm geographically, incidence is more preferable than mortality^11^^)^. Though incidence is currently recorded at many hospitals by themselves, the number of facilities, which are willing to cooperate with the cancer registry system conducted by Ibaraki Prefecture, is regrettably low. Consequently, current Death Certificate Only (DCO) is 44.6% of all malignant neoplasms, 65.2% of pancreas, 52.1% of lung, 45.2% of esophagus, 41.0% of stomach, 37.7% of rectum and colon for combined gender, and 17.4% of breast for females in 1996^12^^)^. Incidence/Death ratio (I/D ratio) is 1.49 of all malignant neoplasms, 1.1 of pancreas, 1.1 of lung, 1.2 of esophagus, 1.6 of stomach, 1.8 of rectum and colon for combined gender, and 3.3 of breast for females in 1996^12^^)^. The report of females breast cancer seems to be well registered because DCO of females breast cancer is low (17.4%). Furthermore I/D ratio of females breast cancer (3.3) is higher than other cancers. It implies that breast cancer prognosis is much better than others. Therefore we would pay attention the difference between incidence and death from malignant neoplasm of better prognosis. One of the reasons of insufficient registration might be that there is little advantage for the majority of physicians who are mainly interested in the effect of treatments. This situation should be improved in the future. Hence, we adopted mortality instead of incidence. Insufficient situation of cancer registration is more likely to be nationwide^13^^)^, with the exception of some prefectures, such as Osaka.

In this study, we attempted to determine whether a geographical differentiation between urbanized and rural areas exists for mortality from malignant neoplasm in Ibaraki Prefecture. When we employ mortality, the differences of medical level concerned with mortality should be considered. The number of medical doctors, belong to medical institutions by medical district, is from 90 to 130 per 100,000 person except Hokota District, 46.7, and thus the difference of medical level could not be large^14^^)^.

The criticism of using SMRs to compare the mortality situation has been discussed in epidemiology since late 1980’s^15^^)^. This means that SMR is not an essential comparative measure because its criteria to compare depend on the population structure in each municipality^16^^-^^19^^)^. Theoretically, the perfect comparable indicator is known as Comparative Mortality Figure (CMF). Though CMF is a perfect comparable measure, it requires mortality of each age stratum in all municipalities and is therefore difficult to employ in a practical sense. On the other hand, SMR is an incomplete measure as a strict sense, but easy to adopt because it needs only the population of each municipality. Fukutomi et al studied comparability and suggested that SMR is a reasonable index for comparison^17^^,^^18^^)^. Takahashi et al clarified a necessary and sufficient condition between CMF and SMR^20^^)^. Furthermore, they evaluated the influence by the distortion away from this condition using actual cancer mortality in all municipalities in Ibaraki prefecture, and also indicated that SMRs are reasonable to use.

Influence of variation of the number of deaths in municipalities with small populations to the cluster structure should be evaluated, because of large variation of SMR. However, to find this effect is not easy, because cluster structure was given visually as a dendrogram, whereas the variation of SMRs could be controlled by variances or confidence intervals. The variation of the number of deaths in all municipalities affects the cluster structure through the least maximum distance, and association with this distance and cluster structure is too complicated to evaluate.

So, to clarify this influence, the simulative comparison between the original cluster and the new cluster through SMR was done. This was obtained by adding one hypothetical dead person to the total number of deaths of each municipality. Consequently, SMRs of each municipality were changed but the structure for hierarchical cluster itself was stable.

The relation of observed clusters and the observed number of death is unknown but interesting problem. When the number of death from malignant neoplasm increases in all municipalities, the pattern of cluster does not change, because the circumstance of mortality between municipalities does not change. However, when the number of death increases in some specified municipality, new cluster is formed by this municipality. For example, Cluster 2 for males consisted of Miwa where SMRs from esophagus cancer were significantly high 3.3 (p<0.01), and thus Miwa formed new cluster.

In the dendrograms resulting from this cluster analysis, the fact that the least maximum distance for males was shorter than for females is an interesting point and worth examining. This difference is based on the mortality structure with SMRs of all sites between municipalities. The shorter distance for males means that combination of males within each cluster is tighter than that of females.

When age-adjusted death rates for malignant neoplasm by site (esophagus, stomach, rectum, liver, pancreas, lung, breast, uterus and leukemia) are compared for ten large cities, other cities and counties in Japan (1990), ten large cities indicate the highest death rate for the adopted sites except leukemia. Particularly esophagus cancer, liver cancer and lung cancer for males and breast cancer for females tend to be higher in urbanized areas. Death rate for leukemia indicates the highest in counties and the lowest in 10 large cities^21^^)^. Therefore, overall rank score of 10 sites would affect the results little.

The relationship between the mortality structure and urbanization was low 0.162 (p=0.14) in males, but a significant correlation, 0.364 (p<0.01), was observed in females. This might be caused by the fact that Japanese females spend more time in their neighborhood than males, and thus females are influenced more by urbanization than males. The above result raises an important epidemiological question, such as: why the structure of mortality from malignant neoplasm for females is influenced by urbanization; and is there any association between females’ lifestyle and mortality from malignant neoplasm? Descriptive epidemiology is needed to reveal further the pattern of mortality from malignant neoplasm.

Dolk et al^22^^)^ proposed that population density be used to discriminate between urbanized and rural areas in their study of disease variation in anophthalmia and microphthalmia in England. They found that a gradient in prevalence from urban to rural area existed and the highest prevalence was observed in the city with the highest population density.

We observed a geographically distinctive distribution for some malignant neoplastic sites. In particular, the municipalities with high mortality from stomach cancer for males tended to be geographically clustered (Figure 3-1), which we believe is due to the similar circumstances of daily life in these neighborhoods. Malignant neoplasm, recently identified as a lifestyle related disease, is the most common cause of death not only in Ibaraki Prefecture, but also in all of Japan.

Salty food and nitrosamine, present in many foods, are suspected of contributing to the risk of stomach cancer. Additionally, the quantity of salt sold is known to be positively correlated with mortality rates for stomach cancer^23^^)^. Corrella et al^24^^)^, in their discussion of the association between dietary habits and geographic pattern of mortality, pointed out that the geographical pattern of mortality from stomach cancer can be explained by variations in food consumption. Furthermore, stomach cancer is thought to be associated with geographical environment^25^^-^^28^^)^. Palmeiro et al^27^^)^ suggested that the geographical distribution of stomach cancer reflects dietary habits, such as production and intake of green vegetables. Facchini et al^28^^)^ described that the geographical differences of stomach cancer might correspond to differences in lifestyle, including dietary habits, rather than to socioeconomic factors. Okubo^29^^)^, and Bradshaw et al^30^^)^ also suggested that stomach cancer is affected by geo-environmental factors. In our research, we found SMRs from stomach cancer for males were high in the southwest part of Ibaraki Prefecture, because there are differences in socio-economic development among the municipalities. This may in turn reflect diversity in general, lifestyle habits.

As esophagus cancer was a potentially distinguishing feature for females, we mapped the areas in which SMRs from esophagus cancer were above 2.0 (Figure 3-2). We were unable to identify clustering in these areas. Although we detected a small clustering of municipalities in which SMRs were 0, we believe that the areas with both high and low SMRs from esophagus cancer are likely distributed throughout Ibaraki Prefecture, because women’s lifestyles might be more similar than men’s in certain areas. Clusters 3 and 5 for females comprised areas with high SMRs from esophagus cancer. The difference between these clusters is SMRs from breast and uterus cancer. There was no notable distinction in Cluster 5, with the exception of high SMRs from esophagus cancer. Cluster 5 consists of the municipalities that were not classified to Cluster 3.

Various dietary habits, on the one hand, are suspected risk factors of esophagus cancer, as in the excessive consumption of alcohol^31^^)^, “Chagayu” (rice gruel cooked in hot tea)^32^^)^, fermented foodstuffs, salty vegetables and cured meat^23^^)^. On the other hand, these risk factors could be associated with nutritional deficiency, consumption of nitrosamines and moldy foodstuff, molybdenum deficiency, poor oral hygiene and specific living habits^33^^)^.

Kano et al used cluster analysis with eight health-related indicators, birth rate, crude death rate, infant mortality, mortality from malignant neoplasm, mortality from heart disease, mortality from cerebrovascular disease, number of hospitals per 100,000 residents, and number of physicians per 100,000 residents. This study revealed that Tsukuba science city, of all municipalities in Ibaraki prefecture, is extremely specified^1^^)^.

Though their study between 1984 and 1986 provided the evidence which Tsukuba science city is extremely different from the other areas, the current study provided less clear evidence. One of the major causes is the different kind of data (only malignant neoplasm Vs nine health-related indicators). However, due to more convenient traffic situation, it is easier for people to go from place to place. This might be another reason for the diminished characteristics.

## CONCLUSION

Cluster analysis formed structure in several distinctive regions for males and females. In the case of the five clusters for males, Cluster 1 includes four municipalities. Three of these are located in the mountainous area of northwest Ibaraki Prefecture, which is characterized by high SMRs from bone marrow. Cluster 5 consists of the mid-south areas, which is characterized by high SMRs from stomach cancer. In the case of the five clusters for females, the clusters seem to be characterized by SMRs from esophagus cancer. A significant association between mortality structure and urbanization is observed for females, but not for males.
